# Preoperative pain treatment in acute abdomen in Osogbo, Nigeria: a randomized double-blind placebo-controlled study

**DOI:** 10.1186/1865-1380-6-3

**Published:** 2013-01-23

**Authors:** Olayide Agodirin, Adetunji Oguntola, Moses Adeoti, Austin Agbakwuru, Kehinde Oluwadiya, Babatunde Olofinbiyi

**Affiliations:** 1 Department of Surgery, LAUTECH Teaching Hospital, Osogbo, Nigeria; 2 Department of Surgery, Obafemi Awolowo University Teaching Hospital Complex, Ile- Ife, Nigeria; 3 Department of Obstetrics and Gyneacology, Obafemi Awolowo University Teaching Hospital Complex, Ile- Ife, Nigeria

**Keywords:** Acute abdomen, Analgesic, Preoperative, Diagnosis, Opioids

## Abstract

**Background:**

Withholding analgesics in acute abdomen for fear of masking clinical features and impairing diagnosis and decision-making is still being practiced despite recent evidence to the contrary. This study assesses the effect of preoperative analgesia on clinical findings, clinical diagnosis, and decision-making in patients with non-trauma acute abdomen.

**Method:**

This is a randomized, double-blind, placebo-controlled study using Tramal, a brand of tramadol, at the ED of LAUTECH Teaching Hospital Osogbo, Nigeria. Ninety-five patients between 18–60 years received Tramal (*n* = 46) or placebo (*n* = 49). The pain score, clinical findings, provisional diagnosis, and treatment plan were noted before and 15–20 min after administration of the analgesic or placebo. The final diagnosis arrived at after adequate investigation or operation was considered the gold standard. The pain scores, diagnosis, treatment plan, and decision between the two groups were compared. Statistical analysis was by SPSS 16. Results were considered statistically significant at *p* < 0.05.

**Results:**

Demography and case distribution were similar in both groups. The improvement in pain was greater in the Tramal group (*p* = 0.001). The abdominal palpation findings were also better in the Tramal group (*p* = 0.02). There were more changes in the diagnosis after use of Tramal (*p* = 0.01). There were more changes in the decision in the Tramal group (*p* = 0.03). Most of the changes in diagnosis and decision in the Tramal group were for the better.

**Conclusion:**

The preoperative use of Tramal in acute abdomen improved the experience of pain and did not adversely affect the accuracy of the diagnosis or decision-making.

## Background

Acute abdomen is the presence of clinical features of intra-abdominal disease that is likely to require surgical intervention for its resolution [1-3]. Because there are numerous causes of acute abdomen, the diagnosis may be a puzzle. Unraveling this puzzle requires obtaining adequate history and examining the patient methodically [3-5]. About 70% of the times, the history and examination, if well conducted, will lead to the diagnosis [1,3].

Pain is often the dominant complaint in patients with acute abdomen [1,3,6,7]. Pain (Latin: poena), true to its Latin meaning “to punishment or torment,” is an unpleasant but protective response to actual or potential tissue damage [8]. In patients with acute abdomen uncontrolled pain may produce an uncooperative patient, thus adding to the difficulty of clinical diagnosis. Uncontrolled abdominal pain may also lead to atelectasis and chest infection from involuntary splinting of respiratory muscles. Tachycardia, hypertension, modification of coagulation and the fibrinolytic system, and lengthy admission are all recognized sequelae of uncontrolled pain [8].

The issue of the safety of providing analgesia for patients with acute abdomen is marked by longstanding controversy over the possible masking of clinical findings and delaying diagnosis [8-10]. In “Early diagnosis of the acute abdomen,” Sir Zachary Cope suggested: “Though it may appear cruel it is really kind to withhold morphine (analgesia) until one is certain or not that surgical interference is necessary” [9]. This reluctance to provide adequate analgesia in acute abdominal pain originated in an era of relative medical underdevelopment when the abdomen was considered a “Pandora’s box” and at a time when morphine was given in doses large enough to sedate patients [8-10]. Since then, many studies have suggested that the administration of analgesics does not hinder accurate diagnosis or treatment and may even be helpful [8,11,12]. Furthermore, increased knowledge about the use of opiates has evolved. Despite this body of knowledge, preoperative analgesia is still avoided in patients with acute abdominal pain for fear of masking the pathology [8,9,13-18]. In keeping with the prediction in the current edition of Cope’s book that old habits may be difficult to kill in the matter of withholding analgesics in patients with acute abdomen [19], in Nigeria the traditional practice of withholding analgesia is still common [10,20].

This study aims at assessing the effect of preoperative analgesia on the clinical findings, diagnosis, and decision-making in patients presenting with non-trauma acute abdomen at the emergency department of our hospital. We hypothesize that preoperative analgesia does not affect the clinical features.

## Methods

This was a prospective, randomized, double-blind placebo study using a synthetic opioid analgesic, the Tramal brand of Tramadol (Grunenthal, Germany), as the active agent and water for injection (WFI) as placebo. It was carried out in the emergency department (ED) of our hospital between October 2008 and May 2010.

The institution’s ethical approval was obtained before commencement of the study, and all randomized patients gave informed consent. All the randomized patients were first seen by the casualty officer, who invited the primary investigator. The primary investigator reviewed the patient’s clinical findings, scored the initial pain (P1) on a numerical rating scale of zero to ten where zero is no pain and ten is the worst pain ever experienced, made a provisional diagnosis (D1), and then proposed the treatment plan (T1).

A third party who was not involved in the patient management presented the patient with an opaque paper bag from which the patient randomly picked an envelope containing the grouping letters ‘A’ for active agent and ‘P’ for the placebo. The third party administered either 2 ml (100 mg) of tramadol (TRA) or 2 ml of water for injection (WFI) intravenously, depending on the group picked by the patient. The group picked and the agent administered were unknown to both the patient and the primary investigator. Fifteen to 20 minutes after the administration of the study agent, the primary investigator reviewed the patient again. The residual pain score was recorded as P2, the new provisional diagnosis as D2, and the new treatment plan as T2. The final diagnosis (D3) was determined postoperatively or after adequate investigation if the patient did not undergo an operation. The definitive treatment (T3) given to the patients was based on the demands of their illness. The diagnosis D3 and the treatment T3 were considered the gold standard against which the D1 and D2 and T1 and T2 were compared. Documentation was stopped for all patients upon discharge from inpatient care. Un-blinding was done after randomization had been closed, and collation and analysis were commenced.

The analysis of treatment, either conservative or operative, and the incision types were based on intention to treat (i.e., patients were considered to be members of whichever treatment group they were originally assigned to regardless of whether or not they took the appropriate or prescribed therapy in part or completely).

The change in clinical diagnosis was considered the primary outcome, and this was used to calculate the sample size because the observed clinical features led to the clinical diagnosis, and the treatment decision was a function of the clinical diagnosis.

In calculating the sample size, a retrospective review of the patients presenting at the ED in the preceding 12 months showed that the accuracy of the provisional diagnosis of all the patients (*N* = 242) managed for acute abdomen was 60% (145/242). This information was used to find the sample size (*n*) for this study using the modified Kirkwood formula for comparison of two groups [21]: *n* = (u + v)^2^{P_1_(1 − P_1_) + P_2_(1 − P_2_)}/(P_1_ − P_2_)^2^.

The desired level of significance, v, for this study was 5% (0.05) v = 1.96 (the two-tailed z value). The chance of detecting an actual difference, that is, the power of the study (u), was set at 75%, 0.67. How large should the difference be between the proportion in one group and the proportion in the other group (P_1_-P_2_) for it to be clinically significant? For this study we took P_1_ as 0.60 [60%, accuracy of clinical (preemptive) diagnosis in the retrospective study] and P_2_ as 0.85 (that is, to achieve a difference of 0.25).

(1) $$n={\left(1.96+0.67\right)}^{2}\left\{0.60\left(1-0.60\right)+0.85\left(1-0.85\right)\right\}/{\left(0.60-0.85\right)}^{2}=40.67\approx 41\text{.}$$

Hence, at least 41 patients were required in each group for this study.

Data analysis was done using SPSS version 16. Statistical analyses were performed using Student’s *t*-test, paired *t*-test (signed ranked), Mann–Whitney U-test, and chi-square or Fisher’s exact test as appropriate. Exclusion criteria included abdominal symptoms more than 7 days before presentation, hypotensive or obtunded patients, inability to comprehend the pain scoring method, reaction to tramadol or opiates, and prior treatment with analgesics in the course of the illness. Patients below 18 and above 60 years were also excluded to ensure homogeneity of the patient pool and dosing of the active agent. During randomization, no attempt was made to match the groups for age, sex, or clinical diagnosis. Randomization was done only when both the primary investigator and the third party were available; this was to eliminate interobserver differences.

## Results

Ninety-five patients aged between 18 and 60 years received either Tramal injection (*n* = 46) or WFI (*n* = 49). The median age in the two groups was 30.0. Males were 54.3% and 47% in the tramadol and placebo groups, respectively. The sex proportions in the two groups were not statistically different (*p* = 0.47).

The distribution of the definitive diagnosis (D3) is shown in Table 1: 75.7% had general surgical conditions, 19.0% medical conditions, 4.2% gynecological conditions, and 1.1% urological conditions. The most frequent diagnosis was acute appendicitis (34.7%). There was no statistically significant difference in the proportion of non-general surgical causes of acute abdomen in the two groups (9/46 in the tramadol group vs. 12/49 in the placebo group, *p* = 0.56). Also the proportion of patients with acute appendicitis in the two groups was not statistically different (*p* = 0.39); see Table 1.

**Table 1:** Distribution of the definitive diagnosis (D3)

	**Definitive diagnosis (D3)**	**Tramadol**	**Placebo**
1	Acute appendicitis	14	19
2	Typhoid ileal perforation	5	1
3	Typhoid enteritis	4	6
4	Adhesive intestinal obstruction	4	3
5	Perforated pud	3	2
6	Acute exacerbation of pud	3	0
7	Perforated appendix	2	2
8	Acute cholecystitis	2	1
9	Sigmoid volvulus	2	1
10	Appendiceal abscess	2	0
11	Appendiceal mass	0	1
12	Anastomotic colonic stricture	0	1
13	Acute pancreatitis	0	1
14	Perforated colonic tumor	0	1
15	Amebic colitis	1	1
16	Infected colonic tumor	0	1
17	Symptomatic ovarian cyst	1	1
18	Peritonism (reduced strangulated hernia)	1	1
19	Pelvic inflammatory disease	1	1
20	Residual abscess	0	1
21	Perforated colon (post-colonoscopy)	0	1
22	Infected renal cyst	0	1
23	Abdominal tuberculosis	0	1
24	Gastroenteritis	0	1
	Total	46	49

**Figure 1:**
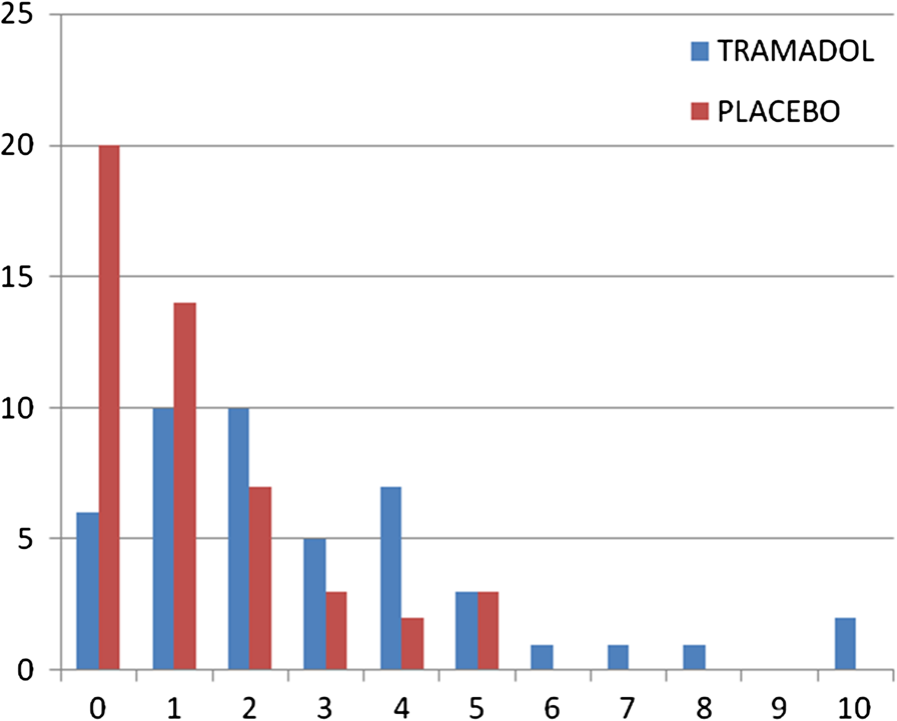
**Frequency of change in pain score (CHP), Mann–Whitney U test (for equality of median). ***P* < 0.001.

The reduction in pain after administration of the study agents is shown in Table 2; the comparison of the change in pain is shown in Figure 1. After administration of Tramal, there was better localization of the abdominal tenderness in 13 patients, and 11 patients were less evasive toward abdominal palpation. In the placebo group, eight had better localization, while six allowed better palpation (*p* = 0.02). After administration of the study agents, there were more cases of discordance between the D1 and D2 in the tramadol group than in the placebo group (10/46 vs. 2/49, *p* = 0.01) (see Table 3). Injection of Tramal made eight of the diagnoses better but induced diagnostic confusion in two patients. WFI made the diagnoses better in two patients; there was no diagnostic confusion in the remaining 47 patients who had no change in diagnosis (see Table 3).

**Table 2:** Change in pain

**Agent**	**Tramadol**		**Placebo**	
**Frequency of pain score**	**P1**	**P2**	**P1**	**P2**
0	-	3	-	-
1	-	3	1	2
2	-	6	5	8
3	-	2	1	7
4	5	9	9	3
5	11	6	9	16
6	1	6	3	3
7	4	6	8	6
8	9	4	5	1
9	6	1	2	2
10	10	-	6	1
Total (*n*)	46	46	49	49
Wilcoxon signed-rank test (for equality of median)	PI > P2		PI > P2	
	*P* < 0.0001		*P* < 0.0001	

**Table 3:** Table of discordance in diagnosis (change in diagnosis)

**Agent**	**D1**	**D2**	**D3**	**Comment**	**Implication**
WFI	APan	APan	ATB	No impact on diagnosis	
WFI	Apmass	Apmass	Ap	No impact on diagnosis	Inappropriate incision
WFI	Apmass	Apmass	Ap	No impact on diagnosis	Inappropriate incision
WFI	PAp	Ap	Ap	Better diagnosis	Better/appropriate incision
WFI	PAp	Ap	Right IRC	Localized clinical signs	Better incision
WFI	PAp	PAp	Ap	No impact on diagnosis	Inappropriate incision
WFI	PAp	PAp	Ap	No impact on diagnosis	Inappropriate incision
WFI	TP	TP	TE	No impact diagnosis	Unnecessary operation
WFI	TP	TP	TE	No impact on diagnosis	Unnecessary operation
TRA	Ap	SOC	SOC	Better diagnosis	Averted unnecessary operation
TRA	Apmass	Ap	Ap	Better diagnosis	Appropriate incision
TRA	Apmass	Ap	Ap	Better diagnosis	Appropriate incision
TRA	ApAbs	Apmass	ApAbs	Diagnostic confusion	Wrong treatment/incision
TRA	PAp	Ap	Ap	Better diagnosis	Better incision
TRA	PAp	Ap	Ap	Better diagnosis	Better incision
TRA	PAp	Ap	PAp	Diagnostic confusion	Wrong incision
TRA	H abs	AC	AC	Better diagnosis	Appropriate treatment
TRA	AIP	No mass	No mass	Better clinical exam	Better diagnosis
TRA	pPUD	aPUD	aPUD	Better diagnosis	Averted unnecessary operation
TRA	TP	TP	PAp	No impact on diagnosis	

Note: *AC,* acute cholecystitis; *AIP*, adhesive inflammatory phlegmon; *Ap*, acute appendicitis; *ApAbs*, appendiceal abscess; *Apan*, acute pancreatitis; *ApMass,* appendiceal abscess; *ATB,* abdominal tuberculosis; *Habs*, hepatic abscess; *IRC*, infected renal cyst; *Pap*, perforated appendix; *pPUD*, perforated PUD; *aPUD*, acute exacerbation of PUD; *SOC*, symptomatic ovarian cyst; *TE*, typhoid enteritis; *TP*, typhoid ileal perforation.

Operative treatment was proposed for 28 and 32 patients after the D1 in the tramadol and placebo groups, respectively. After the administration of tramadol, changes in decision were more frequent (9/28) compared to the placebo group (2/32) (*p* = 0.03).

The rate of complications or appearance of new symptoms was not different: five in the tramadol group and two in the placebo group (*p* = 0.19). All the reported symptoms were non-severe. No patient had respiratory difficulty or hypotension. In the tramadol group 40 patients were discharged, 3 died, and 3 discharged themselves against medical advice (DAMA). Forty-four patients in the placebo group were discharged, and 5 patients died. The median duration of admission was 6 days in both groups.

## Discussion

Several studies indicated inadequate perioperative analgesia and inadequate analgesia for acute and chronic pain worldwide [20,22,23]. In Nigeria, nearly half of our patients, especially non-trauma surgical patients, have no analgesic prescribed prior to definitive surgical intervention [20]. This may be because, coupled with Cope’s pronouncement, the teaching is to treat the cause rather than the symptom, but it is sometimes necessary to administer analgesics preoperatively to mitigate the many untoward effects of uncontrolled pain.

In this study, acute appendicitis was the most common cause of acute abdomen, similar to other documentations, and postoperative adhesion the most common cause of intestinal obstruction. The latter is attributed to the increased rate of laparotomy [22,24-27].

The improvement in pain score after the injection of tramadol being greater than after the use of the placebo is expected. Also, the improvement in abdominal pain demonstrated after the injection of tramadol is similar to the documentation by Mahade et al. on the use of intramuscular tramadol in acute abdominal pain [17]. Some studies involving other opioids have found conflicting results when compared to placebo [8,25].

In this study, in the tramadol group, there was better improvement in the abdominal palpation findings with respect to localization of area of inflammation and disposition of the patients to palpation; the patients were more comfortable and less evasive of abdominal palpation after injection of tramadol. This is an objective confirmation of the subjective pain symptom improvement. The comfort brought to the patient and the acceptance of abdominal examination are very desirable events in the management of patients with acute abdomen because they make gathering information easier. The improvements in pain score and examination findings support the hypothesis that analgesics improve the clinical features against the view that the clinical signs are not affected by pain relief [11]. In this study, however, the placebo effect was also demonstrated. The placebo effect is a psychological effect whereby the believe that an agent has been given to relieve pain and the anticipation of the relief cause release of endogenous opiates, giving the anticipated, desired relieving effect [13,26].

The change in clinical finding in the tramadol group translated into a significant change in the clinical diagnosis in 17% of the patients in this group. The change in diagnosis was for the better more than 75% of the times, but it led to diagnostic confusion in the remainder. Even though this suggests that the use of analgesic has the potential to make the clinical diagnosis better, similar to the report by Zoltie et al. [13], this study may not have given conclusive evidence about this matter. A one-tailed, well-structured, and randomized study may be better to draw unequivocal conclusions. Also, the change in the clinical features and clinical diagnosis led to significant changes in the decisions made regarding operative treatment and incision types in the tramadol group compared to the placebo group. Most of the changes were favorable. Again, it may be tempting to assume that the effect of tramadol translates to better decisions making, resulting in a reduction in inappropriate operations and improvement in the selection of appropriate incision types. Unfortunately, the later assumption may be flawed because of the small number of patients used in that analysis and probably because of the design of this study. However, it is noteworthy that findings are not uniform regarding the effect of preoperative analgesia on the rate of inappropriate operations [7,11,25,27].

The pattern of side effects was similar in the two groups; the side effects were non-severe and not unexpected for the tramadol group. There were no life-threatening events, and the pattern of side effects was similar to that reported by McHale et al. [28] on the use of narcotic analgesics. Although opiates can cause severe adverse effects, this is rare when they are administered in the recommended dose [9,18,23]. This statement is re-affirmed by the pattern of adverse effects in this study.

## Conclusion

This study shows that preoperative use of analgesia in patients with acute abdomen will not mask clinical signs while alleviating the pain experienced by patients. Rather, it may improve the gathering of clinical signs and may potentially help in decision-making.

## Abbreviations

ED: Emergency department; TRA: Tramadol; WFI: Water for injection.

## Competing interests

There are no competing interests. The study was not supported or funded by any organization, institution or individual. The materials used, including the active agent, were funded solely by the authors.

## Authors’ contributions

AS was involved in the conception of the study, design of the study, literature search, collection of data, statistical analysis, drafting of the manuscript, and review of the manuscript. OA was involved in the conception of the topic, design of the study, data analysis, and drafting of the manuscript. AM was involved in the conception of the study, literature search, analysis of the data, drafting of the manuscript, and review. AA was involved in the design of the study, data analysis, and the drafting and review of the manuscript. OK was involved in the design of the study, data collection, statistical analysis, and drafting of the manuscript. OB was involved in data collection, drafting and review of the manuscript. All authors were involved in the review of the available literature and review of the manuscript, and all have agreed that the manuscript be sent to the International Journal of Emergency Medicine for possible publishing.
